# Clinical risk factors associated with radiographic osteoarthritis progression among people with knee pain: a longitudinal study

**DOI:** 10.1186/s13075-021-02540-9

**Published:** 2021-06-04

**Authors:** Milena Simic, Alison R. Harmer, Maria Agaliotis, Lillias Nairn, Lisa Bridgett, Lyn March, Milana Votrubec, John Edmonds, Mark Woodward, Richard Day, Marlene Fransen

**Affiliations:** 1 Faculty of Medicine and Health, Discipline of Physiotherapy, The University of Sydney, Sydney, Australia; 2 Australia Institute of Health Service Management, University of Tasmania, Sydney, Australia; 3 Institute of Bone and Joint Research, The University of Sydney, Royal North Shore Hospital, Sydney, Australia; 4 Graduate School of Medicine, Notre Dame University, Sydney, Australia; 5 St George Hospital Clinical School, University of New South Wales, Sydney, Australia; 6 The George Institute for Global Health, University of New South Wales, Sydney, Australia; 7 The George Institute for Global Health, University of Oxford, Oxford, UK; 8 Department of Epidemiology, Johns Hopkins University, Baltimore, MD USA; 9 Department of Clinical Pharmacology, St Vincent’s Hospital, Sydney, Australia; 10 St Vincent’s Hospital Clinical School, University of New South Wales, Sydney, Australia

**Keywords:** Knee osteoarthritis, Disease progression, Risk factors, Epidemiology, Non-steroidal anti-inflammatory drugs

## Abstract

**Background:**

The aim of this study was to identify modifiable clinical factors associated with radiographic osteoarthritis progression over 1 to 2 years in people with painful medial knee osteoarthritis.

**Methods:**

A longitudinal study was conducted within a randomised controlled trial, the “Long-term Evaluation of Glucosamine Sulfate” (LEGS study). Recruitment occurred in 2007–2009, with 1- and 2-year follow-up assessments by blinded assessors. Community-dwelling people with chronic knee pain (≥4/10) and medial tibiofemoral narrowing (but retaining >2mm medial joint space width) on radiographs were recruited. From 605 participants, follow-up data were available for 498 (82%, mean [sd] age 60 [8] years). Risk factors evaluated at baseline were pain, physical function, use of non-steroidal anti-inflammatory drugs (NSAIDs), statin use, not meeting physical activity guidelines, presence of Heberden’s nodes, history of knee surgery/trauma, and manual occupation. Multivariable logistic regression analysis was conducted adjusting for age, sex, obesity, high blood pressure, allocation to glucosamine and chondroitin treatment, and baseline structural disease severity (Kellgren and Lawrence grade, joint space width, and varus alignment). Radiographic osteoarthritis progression was defined as joint space narrowing ≥0.5mm over 1 to 2 years (latest follow-up used where available).

**Results:**

Radiographic osteoarthritis progression occurred in 58 participants (12%). Clinical factors independently associated with radiographic progression were the use of NSAIDs, adjusted odds ratios (OR) and 95% confidence intervals (CI) 2.05 (95% CI 1.1 to 3.8), and not meeting physical activity guidelines, OR 2.07 (95% CI 0.9 to 4.7).

**Conclusions:**

Among people with mild radiographic knee osteoarthritis, people who use NSAIDs and/or do not meet physical activity guidelines have a greater risk of radiographic osteoarthritis progression.

**Trial registration:**

ClinicalTrials.gov, NCT00513422. This original study trial was registered a priori, on August 8, 2007. The current study hypothesis arose before inspection of the data.

**Supplementary Information:**

The online version contains supplementary material available at 10.1186/s13075-021-02540-9.

## Background

Knee osteoarthritis (OA) is a prevalent chronic joint condition which imposes a significant and increasing burden according to the latest Global Burden of Disease Study [1], and commonly occurs in the medial tibiofemoral compartment [2]. While risk factors for incident knee OA are well understood [3], risk factors for radiographic OA progression are less known and evidence is frequently conflicting [4–6]. Radiographic OA progression is commonly measured by evaluating tibiofemoral joint space narrowing (JSN) on radiographs. The rate of JSN is strongly correlated to cartilage damage on magnetic resonance imaging (MRI) [7] and to knee pain [8] and predicts the need for joint replacement surgery [9]. The rate of joint deterioration varies among individuals, with 57% likely to remain stable, 2% regress, and 41% deteriorate over 15 years [10]. Among people who demonstrate joint deterioration, larger changes in JSN predict future OA-related surgery [9]. Therefore, the ability to identify individuals likely to experience radiographic OA progression is needed to more effectively tailor therapy.

Systematic reviews of risk factors for radiographic OA progression [5, 11] identified strong evidence that greater structural disease severity at baseline (such as marked loss of tibiofemoral joint space width and varus alignment) is associated with radiographic knee OA progression risk. However, there were conflicting findings regarding the risks associated with older age, obesity, past knee injury, pain, and physical function (activity limitation) [5, 11]. Furthermore, it is unknown if people who are physically inactive, using non-steroidal anti-inflammatory drugs (NSAIDs), or using statins (hypothesised to influence cartilage lipid function), are at greater risk of radiographic OA progression. Previous studies have failed to adjust for known factors, such as baseline structural disease severity, which may explain conflicting findings [6, 12].

The aim of this study was to identify modifiable clinical factors that are associated with radiographic OA progression in the medial tibiofemoral compartment. Specifically, we aimed to identify if any modifiable clinical factors are independently associated with medial tibiofemoral compartment JSN over 1 to 2 years in people with symptomatic knee OA, when adjusting for baseline structural disease severity.

## Methods

### Design

A longitudinal study was undertaken as part of a double-blind randomised controlled trial (RCT) evaluating the effect of two dietary supplements (glucosamine sulfate (1500 mg) and/or chondroitin sulfate (800 mg)) on knee OA disease progression over 2 years: Long-term Evaluation of Glucosamine Sulfate (LEGS) study [13]. People with symptomatic knee OA were recruited from the local community through advertisements and primary care centres in New South Wales, Australia, during 2007–2009. All measures (baseline or follow-up) for an individual were collected on a single day at one of four radiology departments. Follow-up assessments were conducted 1 and 2 years later. The study complies with the Declaration of Helsinki. Ethics approval was granted by the Human Research Ethics Committee, University of Sydney (No.8821), and all participants provided written informed consent.

### Participants

Individuals were potentially eligible if they were aged 45–75 years, had knee pain over the past 6 months, had pain on most days of the past month, and rated “worst” pain over the past week as ≥4 on a 0–10 numeric rating scale. They were excluded if they reported having rheumatoid arthritis, bilateral knee replacements, unstable diabetes, allergy to shellfish, lower limb surgery in the past 6 months, intra-articular injections in the past 3 months, or planned knee replacement surgery in the next year [13].

Weight-bearing magnification-controlled posterior-anterior semi-flexed radiographs of both knees were undertaken using a standardised metatarsophalangeal protocol and foot maps [14]. The protocol permitted one repeat, changing the angulation of the beam if the medial tibial inter-rim distance (TIRD) was >2.0mm [15]. Participants were included if a symptomatic knee demonstrated reduced medial tibiofemoral joint space width in comparison with the lateral compartment, but had at least 2.0 mm of joint width.

### Risk factors

Data were collected on age, sex, symptom duration, body mass index (BMI), and waist circumference. Previous self-reported knee trauma or surgery was recorded. Participants’ occupations were classified as manual/physical labour if they reported work in the following areas: technicians/trades, machine operators/drivers, or labourers. Participants were asked to report any regular medication taken during the previous 7 days (including self-prescribed over-the-counter medication) and used to identify NSAID and statin use. Adjustment was made for being allocated to the “double active” intervention group (glucosamine sulfate and chondroitin sulfate; 25% of the cohort) because the results of the RCT demonstrated a reduction in JSN for this allocation compared with placebo [13].

Self-reported knee pain and physical function (activity limitation) were assessed using the Western Ontario and McMaster Universities (WOMAC) OA Index, a reliable and valid disease-specific questionnaire [16]. The WOMAC index (Likert scale) contains subscales for pain (range 0–20) and physical function (range 0–68) where higher scores indicate worse pain or poorer function.

Leisure time physical activity (LTPA) was self-reported using a standardised valid and reliable national questionnaire from the Australian Institute of Health and Welfare [17]. Participants reported activity intensity, frequency, and duration, which was used to determine if physical activity guidelines were met (five sessions, >150 min of activity/week) [18].

Comorbidity was evaluated via the Self-Administered Co-morbidity Questionnaire [19]. The comorbidity questionnaire is scored out of 36, with points allocated for the presence of a condition, treatment, and activity limitation.

For descriptive purposes, health-related quality of life was evaluated using the Medical Outcomes Survey Short Form (SF-12v2) questionnaire, from which two norm-referenced scores were extrapolated: the Physical Component Summary Score (PCS) and the Mental Component Summary Score (MCS) [20], each with a mean (standard deviation (SD)) of 50 (10) in the general population.

### Structural disease severity

Baseline structural disease severity was evaluated using (i) Kellgren and Lawrence (K&L) OA grading scale [21], (ii) minimum medial joint space width, and (iii) tibiofemoral alignment. A single assessor (MF), blinded to treatment but not assessment time point, measured all radiographs for medial compartment joint space width [22] using digitised image analysis software (Holy’s, UCLB, Lyon, France).

Knee alignment was evaluated by measuring the anatomical tibiofemoral alignment from available radiographs and used to calculate the mechanical knee alignment (usually measured by full-length radiographs) using a regression equation [23]. Anatomical alignment axis was the knee joint centre (midpoint of the tibial spine tips) [24], and the angle obtained by drawing 10cm proximal and distal from the centre towards the midshafts of the femur and tibia, respectively. Values of 180° signified neutral alignment, >180° indicated valgus, and <180° indicated varus. A single assessor (MS) conducted all measurements using RadiAnt DICOM Viewer (version 1.9; Medixant, Poland; intra-class correlation coefficient of 20 participants = 0.93, *p* < 0.001).

### Radiographic OA progression

Minimum medial joint space width (mm) was evaluated at all assessment time points. Only radiographs with TIRD of <1.7mm at each time point and ≤0.2-mm difference in TIRD between radiographic evaluations at two time points (baseline, year 1 or 2) were eligible for longitudinal analyses. The primary outcome was medial tibiofemoral JSN ≥0.5mm. The 0.5-mm reduction in JSN is the recommended cut-off value according to the OARSI-OMERACT recommendations, as JSN ≥ 0.5mm over 2 years is considered to indicate a high risk for joint replacement surgery [25] and is a reliable indicator of disease progression [26]. If a follow-up radiograph was unavailable at 2 years, the radiograph at 1 year was used to determine if JSN was ≥0.5mm.

### Statistical analysis

Descriptive statistics such as mean (SD) or number (%) were presented and analyses conducted using SPSS (v22). Potential clinical risk factors which were significantly correlated (*r* > 0.5; *p* < 0.05) were combined into a single factor if they measured a common construct. Of the factors, high BMI (≥30kg/m^2^) and waist circumference (>102cm for men, >88cm for women) were significantly correlated and therefore evaluated as a single obesity risk factor (participants considered obese if at least one factor was present).

All baseline *clinical risk factors* were dichotomized for subsequent analyses. The following factors were investigated: aged > 60years, female sex, obesity (BMI ≥ 30kg/m^2^, or high waist circumference), high blood pressure, presence of Heberden nodes, history of knee surgery/trauma, history of main occupation involving manual/physical labour, use of NSAIDs, use of statins (HMG-CoA reductase inhibitors used for lowering cholesterol), not allocated to “double active” treatment (no treatment of daily combined glucosamine sulfate (1500mg) and chondroitin sulfate (800mg)), high pain at baseline (worst 20% of the cohort on WOMAC (>10/20)), poor physical function (worst 20% of the cohort on WOMAC (>33/68)), and not meeting physical activity guidelines (<150 min of moderate-to-vigorous physical activity/week).

The following *structural disease severity factors* were investigated and included in all models: moderate-to-severe OA (K&L ≥ 2), minimal baseline medial joint space width (the lowest 20% within the cohort, 2.00–3.13mm), and varus alignment (≤178°).

Due to the potential for some known risk factors to be associated with radiographic OA progression, the following variables were chosen a priori to be included as fixed variables in the multivariable model, thus adjusting for age, sex, obesity, high blood pressure [27], high pain at baseline, no glucosamine/chondroitin “double active” treatment, and baseline structural disease severity factors. Univariate associations were examined for all potential clinical risk factors at baseline. Clinical risk factors potentially associated with radiographic OA progression (*p* < 0.2) were then ranked by Pearson χ^2^ values from highest to lowest to determine the order of presentation to a multivariable binary logistic stepwise regression analysis. Variables were retained in the multivariable model if they did not affect model stability (change in the standard error of variables < 20%) and their p-value was < 0.1 [28]. Odds ratios (ORs) and 95% confidence intervals (95% CI) were calculated for each factor associated with radiographic OA progression. Any confirmed clinical risk factor(s) from the multivariable model was then used in exploratory post hoc univariate analyses with all other univariate variables using the χ^2^ test. If medication use was a confirmed risk factor, post hoc analyses were attempted to determine the medication type.

## Results

### Flow of participants

From the 605 participants randomised in the LEGS study, 521 participants attended the radiographic assessment at 1 year, and 484 participants attended the 2-year radiographic assessment. Follow-up radiographs meeting the specifications for assessment (TIRD of <1.7mm at each time point and ≤0.2-mm difference in TIRD between radiographic evaluations) were available for 369 participants (61%) at 2 years, and for an additional 129 participants (21%) at 1 year for whom the 2-year radiographs were not available or of inadequate standard for joint space measurement. Therefore, our analysis was conducted on 498 participants (82% of the sample) who had knee radiographs eligible for longitudinal analysis. There were no differences in baseline characteristics between all participants recruited and those completing follow-up.

Overall, 51% of participants were obese (Table 1), and the mean quality of life scores (particularly PCS) were below the Australian population norms for ages 45–75 years [29]. The low comorbidity score indicates a reasonably healthy sample. The most common comorbidities were back pain (38%), high blood pressure (33%), depression (16%), heart disease (11%), and diabetes (6%). The prevalence of pain was 100% at baseline and 87% at follow-up.

**Table 1:** Participant characteristics at baseline (*n* = 498)

Characteristic	Mean (sd)
Age (years)	60 (8)
Female, N (%)	282 (57%)
Mass (kg)	81 (16)
Height (m)	1.68 (0.95)
BMI (kg/m^2^)	28.7 (5.3)
Waist circumference (cm)	95.1 (13.7)
Females (% with circumference >88 cm)	90.7 (13.6) (52%)
Males (% with circumference >102 cm)	101.0 (11.5) (40%)
Symptom duration (years)	2.5 (1.3)
Medial joint space width (mm)	3.8 (1.0)
OA severity (K&L grade), n (%)	
Grade 1	273 (55%)
Grade 2	202 (41%)
Grade 3	23 (5%)
Knee alignment (°)	181 (2.4)
Knee pain (WOMAC 0–20)	6.7 (3.6)
Physical function (WOMAC 0–68)	21.8 (12.7)
SF 12 PCS	41.4 (9.6)
SF 12 MCS	48.7 (6.8)
Comorbidity score	3.2 (3.0)
Use of non-steroidal anti-inflammatory drugs, n (%)	140 (28%)
Aspirin	49
Meloxicam	34
Ibuprofen	23
Diclofenac	17
Celecoxib	13
Naproxen	8
Indomethacin, ketoprofen, or piroxicam	7

Note: More than one drug was used by ten participants

*N* number, *BMI* body mass index, *OA* osteoarthritis, *K&L* Kellgren and Lawrence, *WOMAC* Western Ontario and McMaster Universities OA index, *SF* Short Form, *PCS* Physical Component Score, *MCS* Mental Component Score

### Factors associated with radiographic OA progression

Radiographic OA progression occurred in 58 participants (12%), of which 47 were evaluated at year 2 and 11 at 1 year. In the univariate analysis, clinical factors which met the criteria for inclusion into the multivariable model were NSAID use, inadequate physical activity, and high baseline pain (Table 2). All indicators of structural disease severity were associated with OA progression (*p* < 0.001).

**Table 2:** Univariate analyses: number (%) with radiographic OA progression, p values for the association between independent variables and radiographic OA progression (JSN ≥ 0.5mm) using Pearson χ^2^

Independent variables	Radiographic OA progression ≥0.5mm at follow-up: n (%)			
	Yes (*n* = 58)	No (*n* = 440)	Unadjusted odds ratio	*p*-value
***Clinical factors***				
Sex (female)	29 (50%)	253 (58%)	0.74 (0.43, 1.28)	0.28
Aged >60 years	34 (59%)	238 (54%)	1.20 (0.69, 2.10)	0.52
Obesity^a^	30 (52%)	225 (51%)	1.02 (0.59, 1.77)	0.93
High blood pressure	17 (29%)	146 (33%)	0.84 (0.46, 1.52)	0.56
Heberden nodes	17 (29%)	130 (30%)	0.99 (0.54, 1.80)	0.97
Manual occupation	11 (19%)	63 (14%)	1.40 (0.69, 2.85)	0.35
Knee trauma or surgery history	24 (41%)	182 (41%)	1.00 (0.57, 1.75)	1.00
No glucosamine/chondroitin	46 (79%)	323 (73%)	1.39 (0.71, 2.71)	0.34
Use of NSAIDs	21 (36%)	119 (27%)	1.53 (0.86, 2.72)	0.14
Use of statins	16 (28%)	115 (26%)	1.08 (0.58, 1.99)	0.81
Inadequate physical activity	50 (86%)	330 (75%)	2.08 (0.96, 4.53)	0.06
High baseline pain (WOMAC pain >10)	8 (14%)	104 (24%)	0.52 (0.24, 1.13)	0.09
Poor physical function (WOMAC PF>33)	9 (16%)	100 (23%)	0.62 (0.30, 1.32)	0.21
***Structural disease severity***				
Disease severity (K&L grade ≥ 2)	39 (67%)	186 (42%)	2.80 (1.57, 5.01)	<0.001
Minimal joint space width (<3.13mm)	27 (47%)	89 (20%)	3.44 (1.95, 6.05)	<0.001
Varus alignment (≤178°)	19 (33%)	54 (12%)	3.45 (1.86, 6.39)	<0.001

^a^Obesity defined as body mass index ≥ 30kg/m^2^ and/or high waist circumference (>102cm for men, >88cm for women)

*NSAIDs* non-steroidal anti-inflammatory drugs, *WOMAC* Western Ontario and McMaster Universities OA index, *PF* Physical Function subscale, *K&L* Kellgren and Lawrence

**Fig. 1:**
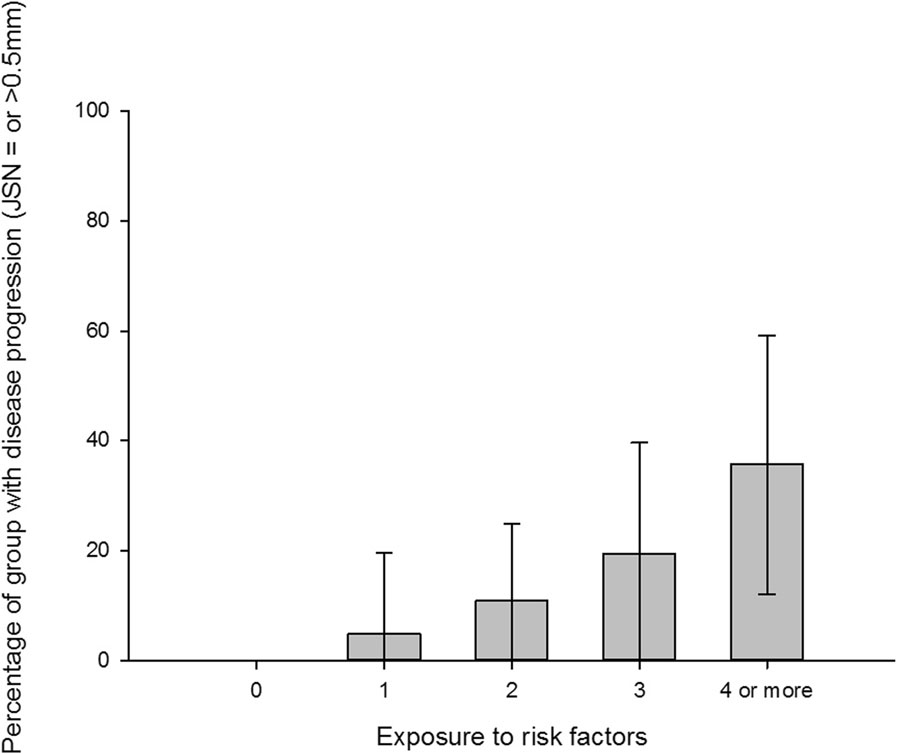
Percentage (95% confidence interval) of participants with radiographic OA progression (JSN ≥ 0.5mm) based on the number of positive risk factors

After adjusting for age, gender, obesity, high blood pressure, no glucosamine/chondroitin supplements, and structural disease severity, people who used NSAIDs at baseline (OR 2.05, 95% CI 1.10–3.84) and people who did not meet physical activity guidelines (OR 2.07, 95% CI 0.92–4.68) were associated with greater odds of radiographic OA progression (Table 3). High baseline pain was no longer significant, but remained in the model as a covariate to adjust for pain. The model explained 16% of the variance in JSN ≥ 0.5mm over 1 to 2 years. Structural disease severity was associated with significantly higher odds of JSN ≥ 0.5mm, including minimal medial joint space width at baseline (OR 2.53, 95% CI 1.31–4.88), varus alignment (OR 2.23, 95% CI 1.09–4.57), and K&L grade ≥ 2 at baseline (OR 1.88, 95% CI 0.98–3.58). The proportion of participants with radiographic OA progression, based on the number of risk factors, is illustrated in Fig. 1.

**Table 3:** Unadjusted and adjusted (multivariable) associations between independent variables (clinical and fixed variables) and radiographic OA progression (JSN ≥ 0 .5mm)

Independent variables	Exposed with progression	Non-exposed with progression	Unadjusted odds ratio (95% CI)	Adjusted odds ratio (95% CI)	***p***-value
***Radiographic OA progression (R*** ^***2***^ ***= 16%)***					
***Clinical factors***					
Use of NSAIDs	15.0%	10.3%	1.53 (0.86, 2.72)	2.05 (1.10, 3.84)	0.025
Inadequate physical activity	13.2%	6.8%	2.08 (0.96, 4.53)	2.07 (0.92, 4.68)	0.080
***Fixed variables***					
Minimal joint space width (<3.13mm)	23.3%	8.1%	3.44 (1.95, 6.05)	2.53 (1.31, 4.88)	0.006
Varus alignment (≤178°)	26.0%	9.3%	3.45 (1.86, 6.39)	2.23 (1.09, 4.57)	0.028
Disease severity (K&L grade ≥2)	17.3%	7.0%	2.80 (1.57, 5.01)	1.88 (0.98, 3.58)	0.056
Age (>60 years)	12.5%	10.6%	1.20 (0.69,2.10)	1.02 (0.56, 1.87)	0.939
Sex (female)	10.3%	13.4%	0.74 (0.43, 1.28)	0.88 (0.48, 1.60)	0.666
Obesity^a^	11.8%	11.5%	1.02 (0.59, 1.77)	1.17 (0.64, 2.14)	0.603
High blood pressure	10.4%	12.2%	0.84 (0.46, 1.52)	0.65 (0.34, 1.25)	0.198
High baseline pain (WOMAC pain >10)	7.1%	13.0%	0.52 (0.24, 1.13)	0.52 (0.23, 1.18)	0.118
No glucosamine/chondroitin combination	12.5%	9.3%	1.39 (0.71, 2.71)	1.26 (0.62, 2.56)	0.521

^a^Obesity defined as body mass index ≥30 kg/m^2^ and/or high waist circumference (>102 cm for men, >88 cm for women)

*NSAIDs* non-steroidal anti-inflammatory drugs, *K&L* Kellgren and Lawrence, *WOMAC* Western Ontario and McMaster Universities OA index

Among participants who used NSAIDs (Table 1), the most predominant were aspirin (35%), meloxicam (24%), ibuprofen (16%), and diclofenac (12%). Due to small numbers, post hoc analyses for each class of NSAIDs used by participants could not be conducted; however, analysis of NSAIDs use without aspirin was performed and yielded similar results. After adjusting for the same factors (age, gender, obesity, high blood pressure, no glucosamine/chondroitin supplements, and structural disease severity), people who used NSAIDs (all except for aspirin) at baseline were associated with greater odds of radiographic OA progression (OR 2.27, 95% CI 1.15–4.46).

Exploratory post hoc χ^2^ tests (Additional file 1) identified that at baseline, a larger proportion of NSAID users in comparison with non-NSAID users had high pain (34% vs 18%, *p* < 0.001), poor physical function (31% vs 18%, *p* = 0.003), and greater reported use of statins (33% vs 24%, *p* = 0.038). A larger proportion of people who did not meet physical activity guidelines, in comparison with those who did, were not allocated to the “double active” glucosamine/chondroitin group (77% vs 65%, *p* = 0.012).

## Discussion

Radiographic OA progression over 1 to 2 years occurred in 58 (12%) participants. Results of this longitudinal study show that people who used NSAIDs and those who did not meet physical activity guidelines independently had double the odds of radiographic knee OA progression compared to people who did not. The study also confirms that people with more severe structural disease at baseline have greater odds of radiographic OA progression. These findings have implications for understanding the pathogenesis of medial knee OA and provide guidance regarding potential interventions for secondary prevention.

Clinical guidelines recommend the use of NSAIDs for people with chronic knee pain unrelieved by simple analgesics [30, 31]. International guidelines also state that NSAIDs should not be recommended for long-term use due to gastrointestinal and cardiovascular adverse effects [32, 33]. While causality cannot be confirmed with this longitudinal cohort study, there may be several reasons why we identified a twofold increased odds of radiographic OA progression among people who reported NSAID use at baseline. Potential mechanisms of elevated progression risk may be due to a physiological effect of the drugs, the representation of people with more severe symptoms, or a representation of people more likely to be physically active due to pharmacological treatment (if activity was proven detrimental to OA). Post hoc analysis provides some support that NSAID users may represent people with greater symptoms, and thus more severe disease. However, this theory is unlikely to fully explain the findings because high baseline pain was not associated with radiographic OA progression in the multivariable analysis. Due to the longitudinal observational nature of the study, we cannot rule out confounding due to potential protopathic bias. Hence, future studies may consider implementing strategies such as a lag-time approach [34]. There is also evidence of a potential physiological effect of NSAIDs from other studies. Other longitudinal observational and RCT studies have identified an increased risk of OA progression in people who use NSAIDs [35], and this may be dependent on the type of NSAID [36]. A placebo-controlled RCT reported that indomethacin use increased progression over a 1-year period, compared with placebo, while tiaprofenic acid did not [37]. Similarly, a longitudinal cohort study of 2330 people with hip or knee OA examined the risk of radiographic OA progression with four common NSAIDs over a mean 7-year follow-up [36]. People who used diclofenac had 2.5-fold increased odds of knee OA progression if diclofenac was taken for 31–180 days, and 3.2-fold increased odds if diclofenac was taken >180 days, whereas ibuprofen, naproxen, and piroxicam were not associated with increased progression. Conversely, cohort data from the Osteoarthritis Initiative (OAI) did not support an increased risk with NSAIDs among their sample of 2890 participants [38]. There may also be support for protective effects of some NSAID classes in other longitudinal studies, with low-dose aspirin use associated with less medial tibial cartilage loss over 2 years compared to non-aspirin users [39]. The high proportion of aspirin use in our study was likely for cardiovascular prophylaxis given the sample was middle aged and overweight and had high blood pressure. A systematic evaluation of the clinical measures of disease progression with different NSAIDs is required for a clearer understanding of potential effects.

People who did not meet guidelines for adequate physical activity had double the odds of radiographic OA progression compared to those who met the guidelines. Due to the observational design, we can only speculate about the causes of the risk. These may include the physiological effect of low physical activity, an association with poor general health or comorbidity, or the presence of more pain or activity limitations in these individuals. Post hoc analysis does not provide support for the abovementioned potential hypotheses. Although a slightly greater proportion of participants not meeting physical activity guidelines were allocated to treatment groups other than the “double active” combined supplements group, thus potentially representing less people in the active therapy group which slowed OA progression in the main trial [13]. This theory is unlikely to explain the findings because treatment allocation was not associated with radiographic OA progression in the multivariable analysis. Despite anecdotal suggestions that high physical activity may be detrimental to OA progression via increased cumulative loading [40], our results suggest that maintaining adequate physical activity is not associated with increased risk and may potentially slow OA progression. Despite the lack of statistical significance in previous studies of physical activity, and a 95% CI which crosses 1.0 in our study, the previously reported mean ORs (range 0.4 to 0.7) suggest agreement with our findings and a potential protective effect of physical activity. Previous studies [6, 12] did not specifically evaluate physical activity duration or intensity at baseline, had smaller cohorts, and did not consider radiographic factors in their analyses. Our findings for the protective effects of physical activity are also consistent with a systematic review which identified that runners had a 50% reduced odds of requiring surgery due to their knee OA compared to non-runners [41]. A mechanism of joint protection is unknown; however, the link between inactivity and chronic low-grade inflammation may explain the identified risk [42]. Muscle strength may present another possible explanation, with high levels of physical activity associated with greater quadriceps strength [43], which is speculated to support the joint with improved shock absorption and load distribution [44]. However, associations between muscle strength and disease progression are conflicting and require further investigation [5, 11]. Studies implementing a physical activity intervention should evaluate effects on joint biology, mediating factors, and OA progression.

The strongest risk factors for radiographic OA progression were baseline structural disease severity measures, indicating that once OA is clearly established on x-ray, OA progression is likely. Our findings are consistent with previous studies which identified similar strengths of association (mean OR range 1.7–4.5) [11]. Varus alignment is also a known factor associated with radiographic knee OA progression, with the current study identifying a twofold increase in odds. This is in agreement with published studies (reported ORs range 2.3–11.0) [11]. The variability in the magnitude of ORs is likely due to different criteria used to measure progression, the angle of varus alignment, and the covariates in the statistical model.

We did not find an association between daily combined glucosamine and chondroitin supplements and the OARSI-OMERACT recommended target for defining disease progression (JSN ≥ 0.5mm over 2 years) [25]. While no effect was identified for rapid progression, the original study confirmed that participants taking the combination supplement experienced a mean of 0.1mm less JSN in comparison to the placebo group over 2 years [13]. This is in agreement with a longitudinal analysis involving the OAI cohort [45], which identified a disease-modifying effect of glucosamine and chondroitin sulfate among people with early OA but not among people with more severe OA. While this benefit has not been shown among people with moderate-to-severe disease [46], the small disease-modifying effect of combined glucosamine and chondroitin during early stages of OA may potentially delay the requirement for joint replacement surgery.

The risk of radiographic OA progression will be higher for people with multiple risk factors. As demonstrated in Fig. 1, 5% of people with only one risk factor demonstrated radiographic OA progression, whereas 36% of people with four or more factors demonstrated progression. Clinicians should routinely assess known risk factors to identify people at the greatest risk of progression and the potential need for joint replacement surgery. Lastly, we did not find an association with age, sex, or obesity, consistent with findings from a systematic review evaluating risk factors of radiographic OA progression [11].

Strengths of this study include the prospective design, consideration of baseline structural disease severity, evaluation of clinically relevant modifiable factors, and participants with predominantly mild radiographic OA at baseline. We also employed a standardised radiographic protocol for image acquisition and evaluation by a single researcher. There are some limitations to be considered when interpreting these findings. As recruitment was for an intervention involving supplements, our results apply to people with symptomatic knee OA seeking treatment. Our analysis was based on self-reported regular NSAID use over the 7 days prior to baseline assessment, which may not reflect continual use of NSAIDs throughout the study. As the study was not powered to separately evaluate the risk of different NSAID classes, future studies should determine if there is a differential effect specific to the class and/or dosage of NSAIDs. However, the significant finding maintained in our post hoc analysis for NSAIDs with the exclusion of aspirin confirms the presence of increased risk. While post hoc analyses can be useful to preliminarily evaluate a hypothesis, it is possible they may lead to chance findings due to low sample sizes and lack of a priori design to answer the specific question. Our main analysis adjusted for nine identified and/or known confounding variables. It is possible that other factors not evaluated may be important to further understand the relationship between NSAID use, physical activity, and radiographic OA progression. With observational longitudinal study designs of radiographic disease progression, there is a possibility of collider bias [47]. As only participants with pre-existing radiographic knee OA were included, conditioning on radiographic OA may bias the effect of risk factors, such as baseline structural disease severity, towards the null effect. Good quality 2-year follow-up radiographs were available for 61% of participants, limiting our ability to detect all people with JSN ≥ 0.5mm. Finally, as this was an observational longitudinal study, risk factors imply an increase in the odds of disease progression, and causality needs to be confirmed using RCT designs.

## Conclusions

Meeting physical activity guidelines should be encouraged among people with symptomatic knee OA with mild structural disease severity, as this may be protective in the risk of radiographic OA progression over 1 to 2 years. Clinicians, and people with knee OA, need to be aware of the increased risk of progression among people who use NSAIDs. The strongest associations with radiographic progression were features of disease severity. Interventions targeting the prevention of radiographic OA progression are likely to be most effective during the early stage of knee OA, when only mild features are evident on radiographs.

## Supplementary Information

**Additional file 1:** Post-hoc univariate analyses of the factors independently associated with radiographic osteoarthritis progression at two years: number (%) of people using NSAIDs and people not meeting physical activity guidelines, respectively. P values for association between independent variables and primary outcomes using Pearson χ^2^.

## Data Availability

The datasets used during the current study are available from the corresponding author on reasonable request.

## Abbreviations

BMI: Body mass index
JSN: Joint space narrowing
K&L: Kellgren and Lawrence OA grading scale
LEGS: Long-term Evaluation of Glucosamine Sulfate study
LTPA: Leisure time physical activity
MCS: Mental Component Summary Score
MRI: Magnetic resonance imaging
NSAIDs: Non-steroidal anti-inflammatory drugs
OA: Osteoarthritis
OR: Odds ratio
PCS: Physical Component Summary Score
RCT: Randomised controlled trial
SD: Standard deviation
SF-12v2: Medical Outcomes Survey Short Form
TIRD: Medial tibial inter-rim distance
WOMAC: Western Ontario and McMaster Universities (WOMAC) OA Index
95%CI: 95% confidence interval
