# Supplementary material for: Clinical risk factors associated with radiographic osteoarthritis progression among people with knee pain: a longitudinal study
Source: Arthritis Res Ther. 2021 Jun 4;23:160. doi: 10.1186/s13075-021-02540-9 (PMC8176608; doi:10.1186/s13075-021-02540-9)
Supplement: Supplementary file 1 — Additional file 1: Post-hoc univariate analyses of the factors independently associated with radiographic osteoarthritis progression at two years: number (%) of people using NSAIDs and people not meeting physical activity guidelines, respectively. P values for association between independent variables and primary outcomes using Pearson χ2. [file 13075_2021_2540_MOESM1_ESM.docx]

Additional file 1: Post-hoc univariate analyses of the factors independently associated with radiographic osteoarthritis progression at two years: number (%) of people using NSAIDs and people not meeting physical activity guidelines, respectively. P values for association between independent variables and primary outcomes using Pearson χ^2^.

Legend:*: Obesity defined as Body Mass Index ≥30kg/m^2^ and/or high waist circumference (>102cm for men, >88cm for women); NSAIDs: Non-steroidal anti-inflammatory drugs; WOMAC: Western Ontario and McMaster Universities (WOMAC) OA index; PF: Physical Function subscale; K&L: Kellgren and Lawrence

| **Independent variables** | **Use of NSAIDs: n (%)** | |  | **Inadequate physical activity: n (%)** | |  |
| --- | --- | --- | --- | --- | --- | --- |
|  | **Yes (n=140)** | **No (n=358)** | **p-value** | **Yes (n=)** | **No (n=)** | **p-value** |
| ***Clinical variables*** |  |  |  |  |  |  |
| Sex (Female) | 79 (56%) | 203 (57%) | 0.96 | 212 (56%) | 70 (59%) | 0.50 |
| Aged >60 years | 82 (59%) | 190 (53%) | 0.27 | 210 (55%) | 62 (53%) | 0.60 |
| Obesity* | 78 (56%) | 177 (49%) | 0.21 | 188 (50%) | 67 (57%) | 0.17 |
| High blood pressure | 54 (39%) | 109 (30%) | 0.08 | 128 (34%) | 35 (30%) | 0.42 |
| Heberden Nodes | 49 (35%) | 98 (27%) | 0.09 | 113 (30%) | 34 (29%) | 0.85 |
| Manual occupation | 15 (11%) | 59 (17%) | 0.10 | 53 (14%) | 21 (18%) | 0.30 |
| Knee trauma or surgery history | 54 (39%) | 152 (43%) | 0.43 | 155 (41%) | 51 (43%) | 0.64 |
| No glucosamine/chondroitin | 99 (71%) | 270 (75%) | 0.28 | 292 (77%) | 77 (65%) | 0.012 |
| Use of NSAIDs | - | - | - | 107 (28%) | 33 (28%) | 0.97 |
| Use of statins | 46 (33%) | 85 (24%) | 0.038 | 98 (26%) | 33 (28%) | 0.64 |
| Inadequate physical activity | 107 (76%) | 273 (76%) | 0.97 | - | - | - |
| High baseline pain (WOMAC pain >10) | 48 (34%) | 64 (18%) | <0.001 | 84 (22%) | 28 (24%) | 0.71 |
| Poor physical function (WOMAC PF>33) | 43 (31%) | 66 (18%) | 0.003 | 79 (21%) | 30 (25%) | 0.29 |
| ***Structural disease severity*** |  |  |  |  |  |  |
| Disease severity (K&L grade≥2) | 58 (41%) | 167 (47%) | 0.29 | 170 (45%) | 55 (47%) | 0.72 |
| Minimal joint space width (<3.13mm) | 26 (19%) | 114 (81%) | 0.12 | 86 (23%) | 30 (25%) | 0.53 |
| Varus alignment (≤178°) | 15 (11%) | 58 (16%) | 0.11 | 56 (45%) | 17 (15%) | 0.97 |
